# The accuracy of ultrasound to predict endotracheal tube size for pediatric patients with congenital scoliosis

**DOI:** 10.1186/s12871-020-01106-7

**Published:** 2020-07-31

**Authors:** Jianhong Hao, Jie Zhang, Buhuai Dong, Zhenguo Luo

**Affiliations:** Department of Anesthesiology, HongHui Hospital, Xi’an JiaoTong University, No.555, YouYi East road, Xi’an, 710054 Shaanxi Province China

**Keywords:** Ultrasonography, Cricoid cartilage diameter, Endotracheal tube, Congenital scoliosis, Pediatric

## Abstract

**Background:**

Ultrasonography has been used to predict the necessary endotracheal tube (ETT) size by measuring the cricoid cartilage diameter. The aim of this study was to determine the accuracy of ultrasound to predict ETT size for pediatric patients with congenital scoliosis.

**Methods:**

Fifty pediatric patients who underwent scoliosis surgery were included in the study. According to the position of the scoliosis, patients were divided into three groups: Group C (cervical lateral bending), Group T (thoracic scoliosis), and Group L (lumbar scoliosis). For all participants, the transverse diameter of the cricoid cartilage was measured with ultrasonography. The initial ETT size was chosen according to the measurements, then the leak test was used to determine the best-fit ETT size. The ETT size predicted by ultrasound and the best-fit ETT size were compared using Bland-Altman analysis.

**Results:**

There was a strong correlation between the best-fit ETT size and the ETT size predicted by ultrasound in Group T (*r* = 0.93, *p* < 0.001) and Group L (*r* = 0.94, *p* < 0.001) and a moderate correlation in Group C (*r* = 0.83, *p* < 0.001). Bland-Altman analysis showed that the ETT size was overestimated by ultrasound in pediatric patients with cervical lateral bending (bias = 0.73 mm, precision = 0.42 mm, limit of agreement = 0.08 to 1.38 mm).

**Conclusion:**

Ultrasound is a reliable tool to predict ETT size for pediatric patients with thoracic or lumbar scoliosis. However, pediatric patients with cervical lateral bending will need an ETT smaller than the size predicted by ultrasonography.

**Trial registration:**

Chinese Clinical Trial Registry, TRN: ChiCTR1900023408, date of registration: 05.26.2019, ‘retrospectively registered’.

## Background

Selection of the optimal endotracheal tube (ETT) size is critical in pediatric anesthesia. A larger-than-optimal-sized ETT can damage the airway [1]. In contrast, a small-sized ETT increases the risk of aspiration and insufficient ventilation [2, 3]. Various methods have been used to estimate the required ETT size, such as age formulas, height formulas, and finger size. However, because of the individual differences, these calculation methods have wide deviations, especially in children [4, 5]. Recent studies have used ultrasonography to predict the optimal ETT size by measuring the cricoid cartilage diameter, and the success rate can reach above 90% [6–8].

Scoliosis is the most common 3-dimensional deformational abnormality of the spine. Rotation of the centrum can produce displacement or rotation of the mainstem bronchi, especially in cervical lateral bending and thoracic scoliosis [9, 10]. Therefore, we conducted this study to assess the ability of ultrasound to accurately predict endotracheal tube size for pediatric patients with cervical lateral bending, thoracic scoliosis, and lumbar scoliosis.

## Methods

The study protocol was approved by the institutional review board of HongHui Hospital. The trial was registered with the Chinese Clinical Trial Registry (ChiCTR: 1900023408). Informed written consent was obtained from the parents of all children.

**Fig. 1:**
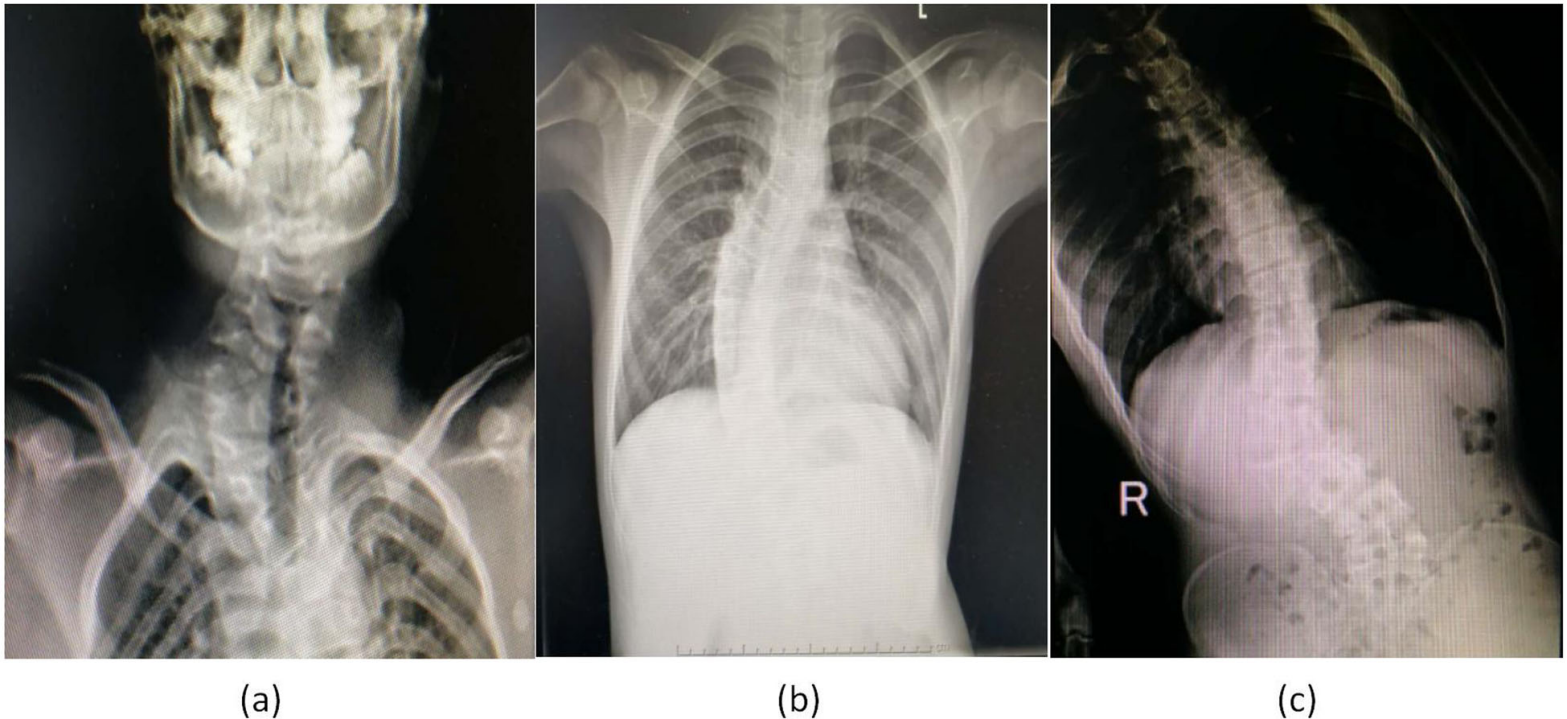
The CT images of the pediatric patients with congenital scoliosis. Cervical lateral bending (**a**); thoracic scoliosis (**b**); lumbar scoliosis (**c**)

Fifty pediatric patients who underwent scoliosis surgery at Hong Hui Hospital, Xi’an Jiaotong University from February 2019 through December 2019 were consecutively enrolled in the study. The inclusion criteria were 1) age 5–12 years and 2) American Society of Anesthesiologists (ASA) physical status I-II. Patients with neck trauma, throat disorders, or an anticipated difficult airway were excluded. According to the location of scoliosis, patients were divided into three groups: Group C (cervical lateral bending), Group T (thoracic scoliosis), and Group L (lumbar scoliosis) (Fig. 1).

**Fig. 2:**
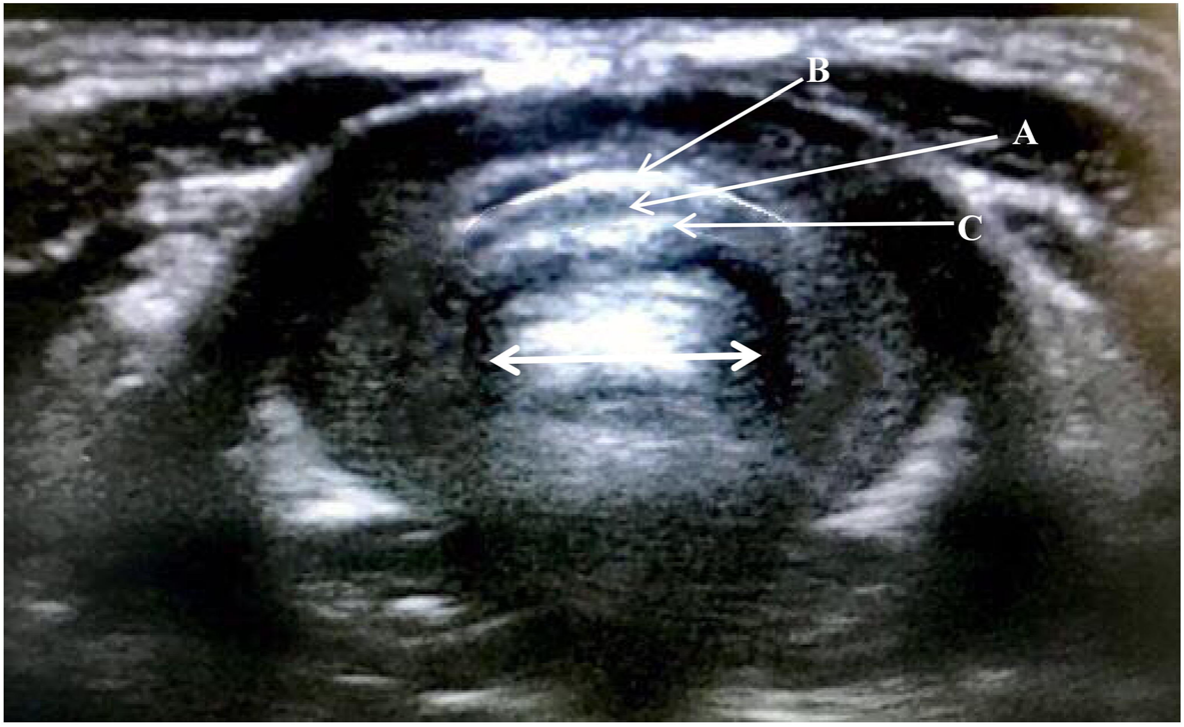
The anatomy of the airway at the level of the cricoid cartilage in the ultrasound image. The cricoid cartilage is a round hypoechoic structure (A) with hypoechoic edges (B and C).The air-column appeared hypoechoic and created a posterior acoustic shadow. The mucosa-air interface created a hypoechoic edge. Solid arrow represents the transverse diameter of the air-column at the level of cricoid cartilage

After transfer into the operating room, non-invasive blood pressure, electrocardiogram, pulse oximetry, end-tidal carbon dioxide concentration, and bispectral index were continuously monitored. The children were administered propofol 1.5 mg^.^kg^− 1^ IV for mild sedation and positioned in horizontal recumbency with slight extension of the head. Ultrasonography using a linear 7–15-MHz probe began with the identification of the true vocal folds. Then the probe was moved caudally to visualize the cricoid arch. The cricoid cartilage appears as a round hypoechoic structure with hyperechoic edges; the air column in the cricoid cartilage appears hyperechoic. The transverse air column diameter was considered to estimate the cricoid cartilage diameter (Fig. 2). Based on the ultrasound measurements of the cricoid cartilage diameter, the corresponding ETT size was selected (Supplemental Table). The measurements of the cricoid cartilage diameter were performed independently by two anesthesiologists, and the average was taken. Before this formal study began, the anesthesiologists had performed 30 measurements of cricoid cartilage diameter under ultrasound guidance.

Anesthesia was induced with fentanyl 4 μg^.^kg^− 1^, propofol 2 mg^.^kg^− 1^, and atracurium 0.4 mg^.^kg^− 1^. After 3 min, tracheal intubation was performed. The leak test was used to determine the best-fit ETT size. In the presence of resistance to passage of the tube into the trachea, or in the absence of an audible leak at airway pressure > 25 cm H_2_O, the ETT was replaced with a tube of 0.5 mm less internal diameter. If a leak was audible at airway pressures < 10 cm H_2_O, if a seal could not be achieved with a cuff pressure > 25 cm H_2_O, or if a peak airway pressure < 25 cm H_2_O was observed during ventilation, the tube was exchanged for one a size larger. The ETT size predicted by ultrasonography and the best-fit ETT size were recorded for each patient. The leak test and the tracheal intubation were performed by another investigator who was blinded to the cricoid cartilage diameter measurements.

Anesthesia was maintained with remifentanil (0.16 μg^.^kg^-1.^min^− 1^) and sevoflurane (1.5%). At the end of all surgical procedures, sevoflurane and remifentanil infusion was stopped. The trachea was extubated when spontaneous ventilation returned. After extubation, the children were transferred to the PACU. The number of patients with laryngospasm was recorded.

The data were analyzed using SPSS® v. 18 for Windows (IBM, Inc., Armonk, NY, USA). The demographic characteristics were compared using the chi-squared test or ANOVA. A scatter plot between the best-fit ETT size and the ETT size predicted by ultrasound was constructed. A Bland-Altman plot was generated to analyze the agreement between the ETT size predicted by ultrasonography and the best-fit ETT size. A *P* value < 0.05 was considered statistically significant.

## Results

Fifty pediatric patients with congenital scoliosis were included in this study. No significant differences were found in age, sex, height, and weight between the three groups (Table 1).

**Table 1:** Demographic characteristics

Group	C (*n* = 13)	T (*n* = 26)	L (*n* = 11)
Age (years)	7.5 (1.6)	9.0 (2.0)	8.5 (1.8)
Sex (male/female)	5/8	12/14	4/7
Hight (cm)	125.2 (10.8)	130.4 (15.7)	130.8 (14.0)
Weight (kg)	23.2 (6.5)	27.8 (10.4)	19.5 (14.1)

Data are mean (SD) or ratio. *C* Cervical lateral bending, *T* Thoracic scoliosis, *L* Lumbar scoliosis

**Fig. 3:**
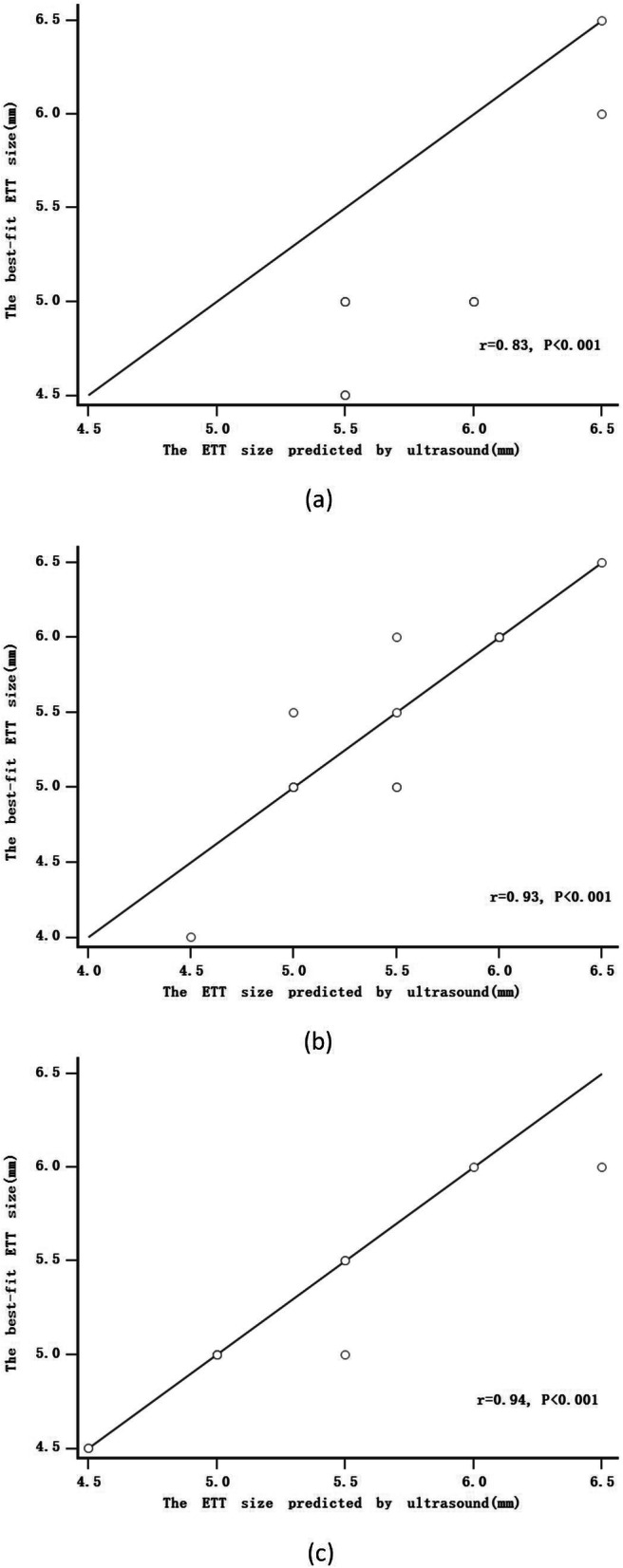
The scatter plot of the the best-fit ETT size and the ETT size predicted by the ultrasound in Group C (**a**), Group T (**b**) and Group L (**c**)

There were strong correlations between the best-fit ETT size and the ETT size predicted by ultrasound in Group T (*r* = 0.93, *p* < 0.001; Fig. 3b) and Group L (*r* = 0.94, *p* < 0.001; Fig. 3c) and a moderate correlation with Group C (*r* = 0.83, *p* < 0.001, Fig. 3a).

**Fig. 4:**
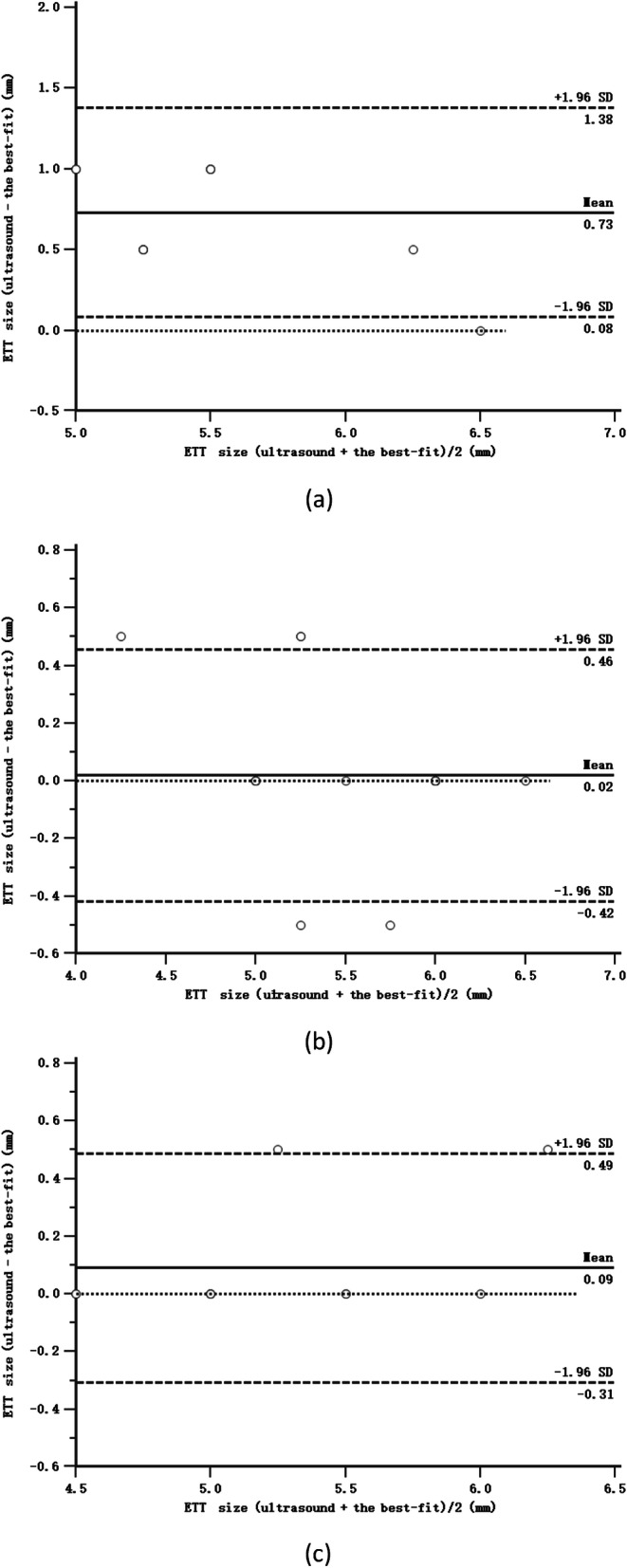
Bland-Altman graph of the best-fit ETT seze and the ETT size predicted by the ultrasound in Group C (**a**), Group T (**b**) and Group L (**c**)

Bland-Altman analysis showed no obvious bias between the ETT size predicted by ultrasonography and the best-fit ETT size in Group T (bias = 0.02 mm, precision = 0.12 mm, limit of agreement = − 0.42 to 0.46 mm; Fig. 4b) and in Group L (bias = 0.09 mm, precision = 0.17 mm, limit of agreement = − 0.31 to 0.49 mm; Fig. 4c), but the ETT size was overestimated by ultrasound in Group C (bias = 0.73 mm, precision = 0.42 mm, limit of agreement = 0.08 to 1.38 mm; Fig. 4a).

Compared with Group T and Group L, the incidence of laryngospasm was higher in Group C (15.0% vs 3.8%, *p* < 0.05; and 15.0% vs 0.0%, *p* < 0.05, respectively).

## Discussion

The cricoid cartilage, as the narrowest part of the larynx in children, plays an important role in the selection of optimal ETT size for intubation [11, 12]. However, recently, Dalal et al. [13] found that the vocal cord and subvocal cord areas were the narrowest portion in pediatric airways. Compared with the vocal cords, the cricoid is a complete and relatively rigid cartilaginous ring and the most frequently damaged structure during endotracheal intubation [14]. Therefore, theoretically, the cricoid cartilage is the limiting factor during intubation and can be a predictive factor in the selection of optimal ETT size for intubation.

Ultrasonography is safe, noninvasive, can be quickly performed, provides real-time images, and is relatively simple to learn. More importantly, the leading edge of the cricoid cartilage and the air column can be identified with ultrasound [15]. In our study, the cricoid cartilage diameters were estimated from the measurements of the transverse air column diameter, which is the most common approach to measure the transverse diameter of the cricoid cartilage in clinical research. To ensure the accuracy of the measurements, they were performed independently by two anesthesiologists and the average was taken, and the anesthesiologists who performed ultrasound examinations had performed 30 procedures before the formal experiment began. Lakhal et al. [16] found that 15 procedures were enough for an operator to obtain reliable measurements. Moreover, Julio et al. found that subglottic diameter ultrasound measurements had high intra-rater and inter-rater reproducibility [17]. Therefore, we believe that our measurements were accurate.

The leak test is a classic experimental method and has been applied to determine the best-fit ETT size for many years. Therefore, in our study, best-fit ETT size was chosen according to the leak test. In prior studies, the allowed leak pressure often was 15–30 cm H_2_O for cuffed ETT [18]. Scoliosis can affect pulmonary function, and lung function abnormalities are mainly of the restrictive type [19]. During the procedure, the children are positioned in prone recumbency, and the operation can apply pressure to the chest. All these factors can cause elevation of the airway pressure. Therefore, we chose a higher leak pressure of 25 cm H_2_O to determine the best-fit ETT size.

Our study showed a strong correlation between the ETT size predicted by ultrasonography and the best-fit ETT size in pediatric patients with thoracic or lumbar scoliosis. The Bland-Altman analysis showed no obvious bias between the ETT size predicted by ultrasonography and the best-fit ETT size in pediatric patients with thoracic or lumbar scoliosis. Our findings were consistent with those reported by Pillai et al. [6]. In their study, the correlation was 0.98, and the bias was 0.041 mm. Therefore, it is feasible to predict ETT size by measuring the transverse diameter of the cricoid cartilage with ultrasonography in pediatric patients with thoracic or lumbar scoliosis.

However, in pediatric patients with cervical lateral bending, there was a poor correlation between the ETT size predicted by ultrasonography and the best-fit ETT size. Bland-Altman analysis showed that the average of the differences between the ETT sizes was 0.73 mm. Considering that the minimal increment for tube size change is 0.5 mm (according to the inner diameter of the ETT), the ETT size was overestimated by ultrasound in pediatric patients with cervical lateral bending.

**Fig. 5:**
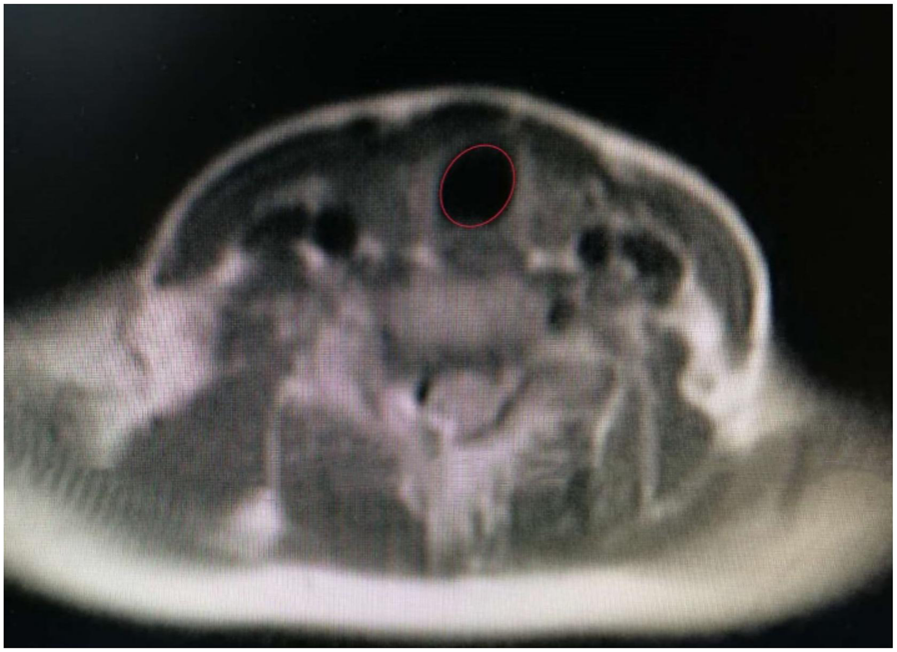
The anatomy of the neck of the pediatric patients with cervical lateral bending in MRI. The red circle represents the cricoid cartilage

MRI is considered the reference standard for evaluating the larynx. High-quality images of the cricoid cartilage can be acquired by MRI, and the cricoid cartilage diameter can be accurately measured [20]. Therefore, in order to ascertain the cause of our result for Group C, we reviewed MRI images of four patients with cervical lateral bending. We found that the cricoid cartilage of the patients with cervical lateral bending was rotated (Fig. 5). Previous research has shown that rotation of the centrum can produce displacement or rotation of the mainstem bronchi [9, 10]. Therefore, we speculate that the rotation of the cricoid cartilage results from deviation or rotation of the cervical vertebrae.

**Fig. 6:**
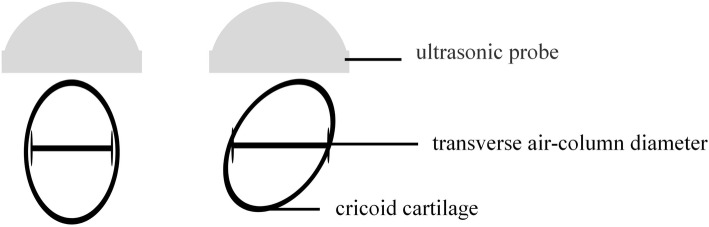
The schematic diagram of the measurements if the cricoid cartilage diameter by ultrasonography in pediatric patients with cervical lateral bending. The figure was generated using Microsoft Office 2013, and was not under copyright

Under normal circumstances, the cricoid cartilage is elliptical, and the transverse diameter is smaller than the anteroposterior diameter [21]. When measuring the transverse diameter of the cricoid cartilage by ultrasound, the probe is positioned on the anterior neck, and the transverse air column diameter is measured to estimate the cricoid cartilage diameter. Because of this, rotation of the cricoid cartilage can broaden the air column as measured by ultrasound, resulting in a larger diameter measurement and overestimation of the necessary ETT size (Fig. 6). In our study, we found that the ETT size predicted by ultrasonography was larger than the best-fit ETT size in pediatric patients with cervical lateral bending. Therefore, compared to what is predicted by ultrasonography, these patients need a smaller ETT.

The incidence of laryngospasm was higher in the patients with cervical lateral bending. There are two possible reasons for this result. First, in pediatric patients with cervical lateral bending, the initial ETT size predicted by ultrasound was larger, and a larger ETT can irritate the throat and increase the likelihood of laryngospasm. Second, operation on the neck can irritate the upper airway, also increasing the likelihood of laryngospasm.

The present study has one limitation. Only four patients with cervical lateral bending underwent cervical MRI; therefore, it is not entirely clear whether the deviation of ultrasonographic measurements resulted only from the rotation of the cricoid cartilage in these patients. Further study is needed to analyze the influence of cricoid cartilage morphology on the accuracy of ultrasound to measure the cricoid cartilage diameter in pediatric patients with cervical lateral bending.

## Conclusion

Ultrasound is a reliable tool to predict ETT size for pediatric patients with thoracic or lumbar scoliosis. However, compared to the ETT size predicted by ultrasonography, pediatric patients with cervical lateral bending need a smaller ETT.

## Supplementary information

**Additional file 1: Table S1.** Outer and inner diameters of cuffed endotracheal tubes of the used brand*.

## Data Availability

The datasets used and analyzed during the current study are available from the corresponding author on reasonable request.

## Abbreviations

ETT: Endotracheal tube
ASA: American Society of Anesthesiologists
PACU: Post-anesthesia care unit
ANOVA: Analysis of variance
MRI: Magnetic resonance imaging
