# Supplementary material for: The accuracy of ultrasound to predict endotracheal tube size for pediatric patients with congenital scoliosis
Source: BMC Anesthesiol. 2020 Jul 31;20:183. doi: 10.1186/s12871-020-01106-7 (PMC7394693; doi:10.1186/s12871-020-01106-7)
Supplement: Supplementary file 1 — Additional file 1: Table S1. Outer and inner diameters of cuffed endotracheal tubes of the used brand*. [file 12871_2020_1106_MOESM1_ESM.docx]

**Supplemental Table Outer and inner diameters of cuffed endotracheal tubes of the used brand*.**

| CCD  (mm) | >3.3 | 3.4-4.0 | 4.1-5.3 | 5.4-6.0 | 6.1-6.7 | 6.8-7.3 | 7.4-8.0 | 8.1-8.7 | 8.8-9.3 | 9.4-10.0 | <10.1 |
| --- | --- | --- | --- | --- | --- | --- | --- | --- | --- | --- | --- |
| OD  (mm) | 3.3 | 4.0 | 5.3 | 6.0 | 6.7 | 7.3 | 8.0 | 8.7 | 9.3 | 10.0 | 10.7 |
| ID  (mm) | 2.0 | 2.5 | 3.5 | 4.0 | 4.5 | 5.0 | 5.5 | 6.0 | 6.5 | 7.0 | 7.5 |
| *Xinxiang Tuoren Medical Equipment Co., Ltd, Henan, China.  CCD: Cricoid cartilage diameter; OD: outer diameter; ID: inner diameter | | | | | | | | | | | |
